# The clinical course of untreated CIN2 (HPV16/18+) under active monitoring: A protocol of systematic reviews and meta-analysis

**DOI:** 10.1097/MD.0000000000032855

**Published:** 2023-02-10

**Authors:** Buwei Han, Mengke Yuan, Yi Gong, Ding Qi, Tong Jiang, Jian Li, Yiming Sun, Li Liu

**Affiliations:** a Heilongjiang University of Chinese Medicine, Harbin, People’s Republic of China; b Beilun Branch, The First Affiliated Hospital of Zhejiang University Medical College, Ningbo, People’s Republic of China; c Affiliated Hospital of Guizhou Medical University, Guiyang, China; d Heilongjiang Academy of Traditional Chinese Medicine, Harbin, People’s Republic of China; e Department of Gynecology, The First Affiliated Hospital, Heilongjiang University of Chinese Medicine, Harbin, People’s Republic of China.

**Keywords:** cervical cancer, conservative treatment, grade 2 cervical intraepithelial neoplasia, human papillomavirus

## Abstract

Cervical cancer (CC) is one of the most prevalent and fatal cancers among women. Nearly all forms of CC are related to HPV, and 70% of invasive CCs are associated with HPV16 and HPV18. A histologically confirmed case of cervical intraepithelial neoplasia (CIN)2 or a more severe histological diagnosis is considered to be the demarcation point for treatment, but overtreatment will increases the risk of preterm birth in subsequent pregnancies. This study will evaluate the progress of CIN2 (progression, persistence, or regression) in HPV16/18^+^ CIN2 patients who were managed conservatively for 3 months.

**Methods:**

PubMed, Cochrane Library, China National Knowledge Infrastructure, Cumulative Index for Nursing and Allied Health Literature (CINAHL), and the Excerpta Medica Database will be searched. We will include studies reporting on women with CIN2 and HPV16/18^+^, conservative treatment for 3 to 60 months with disease outcomes including progression (CIN3 or worse), persistence (CIN2), and regression rates (CIN1 or less). The primary outcome will be the progress of CIN2. Two authors will search the relevant literature, extract the data, and assess the risk of bias. A funnel chart will be used to identify publication or other reporting biases, and the AHRQ guidelines will be used to assess the risk of bias in each included study. The *I*^2^ statistic will be used to assess heterogeneity. If there is a high degree of heterogeneity between the studies, the random effects model will be used; otherwise, a fixed effects model will be used.

**Results:**

The results of this systematic review will be published in a peer-reviewed journal.

**Conclusion:**

This systematic review will evaluate the clinical development of patients with conservatively monitored histologically confirmed HPV16/18+ CIN2.

## 1. Introduction

Cervical cancer (CC) is one of the most prevalent and fatal tumors among women.^[1–3]^ Each year, over half a million CC patients are diagnosed worldwide, and there are over 300,000 deaths as a direct result of CC. Death from CC is 18 times more common in low- and middle-income regions than in developed regions, according to a study that looked at the lack of regular screening and HPV vaccination in low- and middle-income regions, meaning that the disease is largely preventable.^[4]^ Nearly types of CC, including adenocarcinoma, glandular squamous cell carcinoma, and squamous cell carcinoma, are linked to human papillomavirus (HPV) infection.^[5,6]^ HPV infection is a widespread infectious disease, with women having a lifetime risk of infection exceeding 80%.^[7]^ So far, over 140 HPV genotypes have been identified.^[8]^ Among them, 40 subtypes related to reproductive tract infection can be categorized into high-risk and low-risk subtypes in accordance with different levels of carcinogenicity. Infections with high-risk HPV subtypes are prone to develop into high-level squamous intraepithelial lesions and even CC.^[9,10]^ HPV16 and HPV18 are associated with 70% of all cases of invasive CC, and HPV16 is the most common high-risk subtype, causing over half of all CC cases.^[11,12]^ In 2012, the American Society for Colposcopy and Cervical Lesions and the American College of Obstetricians and Gynecologists issued guidelines clearly stating that people infected with HPV16 and HPV18 should undergo colposcopy even if cytology is negative.^[13]^ However, fewer than 10% of patients with high-risk HPV subtypes have an infection lasting more than 2 years. Rational clinical decision making can avoid wasting medical resources and alleviate pain in patients.

HPV infection is a sexually transmitted disease that causes cervical intraepithelial neoplasia (CIN) and precedes CC. Currently, we consider CIN1 a benign lesion that is best managed conservatively, whereas CIN3 is considered a true precursor of invasion and may lead to further deterioration. The clinical processes and biological performance of CIN2 remain unclear. The histological diagnosis of CIN2 is generally considered the threshold for physicians to choose whether to proceed with treatment. CIN2 is a vague histological diagnosis with a high rate of spontaneous regression.^[14]^ Local cervical excision is usually performed clinically to treat CIN3 or even CIN2.^[15]^ Although treatment has been shown to be effective, it increases the risk of miscarriage in the first trimester and second trimesters.^[16–18]^ Finding ways to avoid unnecessary overtreatment is important in women of childbearing age. In some studies, the high rate of regression and treatment-related morbidity of CIN2 has led to alternative conservative treatment strategies in adolescents and young women. A 2018 analysis of high-quality studies recommended that women with histologically proven CIN2 be actively monitored for conservative treatment.^[19]^ It is clear that HPV16/18 is the most prevalent HPV type associated with CC,^[20]^ leading us to question the population scope of this recommendation.

However, there has been no systematic review of the clinical development of patients with conservatively monitored histologically confirmed HPV16/18^+^ CIN2. This study will evaluate CIN2 progression (progression, persistence, and regression) in patients with HPV16/18^+^ CIN2 and > 3 months of conservative treatment to provide reasonable recommendations for future clinical decision-making.

## 2. Objective

Our study aims to evaluate the incidence of CIN2 progression (CIN3 or worse), persistence (CIN2), and regression (CIN1 or less) in patients with HPV16/18^+^ CIN2 at different follow-up time points (3–60 months).

## 3. Methods and analysis

Stata16 software will be used to conduct the meta-analysis, which will comply with the Cochrane Collaboration Manual and PRISMA Statement Guidelines to provide an account of the results.^[21]^

### 3.1. Eligibility criteria considering studies for this review

#### 1.3.1. Study design.

This systematic review will include all forms of research including randomized controlled trials, non-randomized controlled studies, case-control studies, and case-cohort studies, in any country or region and published in any language.

#### 2.3.1. Participants.

Non-pregnant adult women (≥18 years old) with a histological diagnosis of CIN2 and positive cervical HPV16/18 test results will be included, regardless of ethnicity or education. Studies using histology will be preferred to cytology for the diagnosis of disease grade during the follow-up period; however, cytological examination is also accepted if histology is not available, especially in the case of normal findings. We will exclude studies with follow-up results of less than 3 months or greater than 60 months.

#### 3.3.1. Interventions.

Studies including patients without any related medical treatments but conservatively managed for 3 to 60 months will be included.

#### 4.3.1. Outcomes measures.

Main outcomes: The rate of progression to CIN3 or invasive CC, persistence CIN2 and regression (CIN1 or less) rates, and the default rate of HPV16/18^+^ CIN2 at different follow-up time points (3–60 months). Secondary outcomes: rates of bleeding complications, infectious diseases, and adverse pregnancy outcomes.

### 3.2. Data collection and analysis

#### 1.3.2. Information sources.

Under the premise of not being critical of the publication status or language of the research, we will comprehensively search the PubMed, Cochrane library, Cumulative index for nursing and allied health literature (CINAHL), excerpta medica database, and China national knowledge infrastructure databases with MeSH terms–(“human papilloma virus 16” or “HPV16” or “human papilloma virus 18” or “HPV18”) and (“CIN2”) and (“disease progression” or “progress”). Our search will be limited to studies published after January 1, 1973 (when the CIN classification was introduced).^[22]^

#### 2.3.2. Selection of studies.

**Figure 1.**
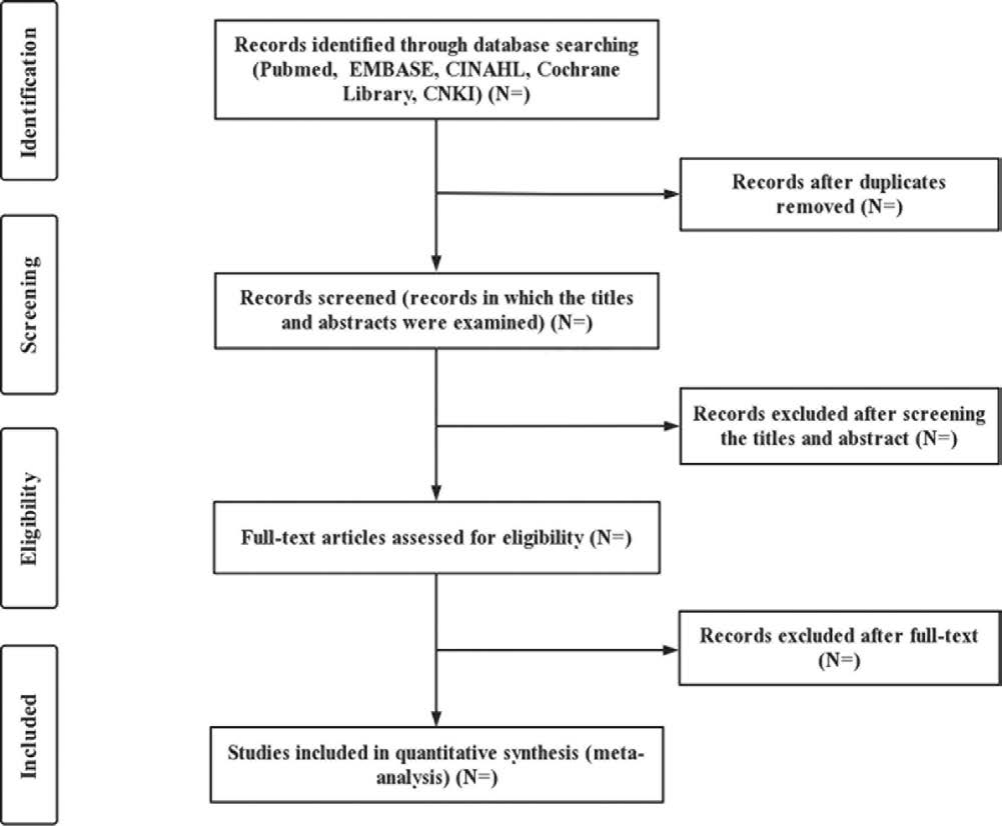
Process for selecting articles.

These included articles will be entered into Endnotes 9 software for unified management, and the title, abstract, etc. will be further searched according to the inclusion criteria. Potential articles will be read in full to determine whether they should be included, and the first two authors (BWH and MKY) will conduct independent searches. The articles included in the study will be subjected to data extraction, which includes the author, publication date, article type, sample size, average age of the subjects, number of positive progressions of HPV (16/18)^+^ CIN2, and number of HPV (16/18)^+^ CIN2 cases. Other methodological characteristics will be included, as deemed necessary. If there is a dispute about the included literature or data extraction, it will be decided after discussion with a third researcher (YG). The process of selecting the articles is shown in the PRISMA flowchart (Fig. 1).

#### 3.3.2. Assessment of the risk of bias.

The researchers (BWH and MKY) will use the Agency for Healthcare Research and Quality^[23]^ guidelines to assess the risk of bias in each incorporated study. The risk of bias assessment will be divided into selection, implementation, follow-up, measurement, and reporting biases. Eleven items will be included, and these will be evaluated as yes, no, or unclear. Disputed issues will be discussed with the third author (YG) and a final decision will be made.

#### 4.3.2. Assessment of reporting biases.

A funnel chart will be used to identify publication bias or other reporting biases. If the funnel chart shows publication bias (*P* < .05), subgroup or sensitivity analysis will be used to further evaluate the source of bias. The research results will be analyzed carefully.

#### 5.3.2. Assessment of heterogeneity.

Th evaluate article heterogeneity, we will choose the *I*^2^ statistic to check for heterogeneity and appraise inconsistency in all selected studies. If *I*^2^ < 25%, no heterogeneity will be considered; if *I*^2^ is between 25% and 50%, the degree of heterogeneity will be considered minimal; if *I*^2^ is between 50% and 75%, the heterogeneity will be considered medium; and if *I*^2^ > 75%, the degree of heterogeneity will be considered high. If there is a high degree of heterogeneity between the studies, the random effects model will be used; otherwise, a fixed effects model will be used.

#### 6.3.2. Data synthesis and statistical analysis.

In agreement with the guidelines formulated by the Cochrane Collaboration, this study will use Stata Version 16 software (Stata Corp, College Station, TX) to perform a meta-analysis of the extracted data. Categorical variables are expressed as risk ratios with 95% confidence intervals (CIs). When the extracted data are determined, we will use email to contact the original author, if possible, to ensure the accuracy of the data and overall research results. All studies will be performed using the intent-to-treat principle. When *I*^2^ < 75%, we will use a fixed-effects or random-effects model.

#### 7.3.2. Subgroup analysis and sensitivity analysis.

The investigation of subgroups according to age (≤30 years and >30 years) will be planned, and we will evaluate the sensitivity of the study to ensure the robustness of each individual study.

#### 8.3.2. Confidence in the cumulative evidence.

Our study will assess the weight of outcome evidence using a methodology called Grading of Recommendations Assessment, Development, and Evaluation.^[24]^ The quality of the evidence will be evaluated based on five factors: study limitations, effect consistency, inaccuracy, indirectness, and publication bias. The results will be classified as high-, medium-, low-, or very low-quality.

#### 9.3.2. Patient and public involvement.

No patient involved.

## 4. Ethics and dissemination

Ethical approval is not required for our study because we do not use personal data. We will publish our findings in peer-reviewed publications and journals, or disseminate them publicly.

## 5. Strengths and limitations

Our study will more comprehensively determine the incidence of CIN2 progression, persistence, and regression as well as the default incidence in patients with HPV16/18^+^ CIN2 at different follow-up points (3–60 months). We will not restrict the language when conducting a comprehensive systematic search of the electronic databases, and will also check the reference sections of the selected articles for relevant papers. The goal of this study is to identify high-quality studies. However, the clinical course and biological properties of CIN2 remain unclear, and the classification of CIN based on the epithelial thickness of cervical lesions possesses certain subjective differences among observers, which may lead to high heterogeneity between studies and hinder our assessment of study quality.

## Acknowledgment

We would like to thank our department librarian for reviewing the schema and helping with the search strategy.

## Author contributions

**Conceptualization:** Buwei Han, Mengke Yuan, Yi Gong.

**Formal analysis:** Buwei Han, Mengke Yuan, Yi Gong.

**Funding acquisition:** Li Liu.

**Investigation:** Buwei Han, Mengke Yuan, Yi Gong, Ding Qi, Tong Jiang, Jian Li.

**Methodology:** Buwei Han, Mengke Yuan, Yi Gong, Ding Qi, Tong Jiang, Jian Li.

**Project administration:** Yiming Sun, Li Liu.

**Resources:** Li Liu.

**Supervision:** Yiming Sun, Li Liu.

**Validation:** Yiming Sun, Li Liu.

**Visualization:** Ding Qi.

**Writing – original draft:** Mengke Yuan.

**Writing – review & editing:** Buwei Han.
